# The Effect of Acknowledged and Novel Anti-Rheumatic Therapies on Periodontal Tissues—A Narrative Review

**DOI:** 10.3390/ph14121209

**Published:** 2021-11-23

**Authors:** Maria-Alexandra Martu, George-Alexandru Maftei, Ionut Luchian, Ovidiu Mihail Stefanescu, Mihaela Monica Scutariu, Sorina Mihaela Solomon

**Affiliations:** 1 Periodontology Department, Faculty of Dental Medicine, “Grigore T. Popa” University of Medicine and Pharmacy, 16 Universitatii Str., 700115 Iasi, Romania; maria-alexandra.martu@umfiasi.ro (M.-A.M.); sorina.solomon@umfiasi.ro (S.M.S.); 2 Oral Pathology Department, Faculty of Dental Medicine, “Grigore T. Popa” University of Medicine and Pharmacy, 16 Universitatii Str., 700115 Iasi, Romania; 3 Dento-Alveolar Surgery and Anesthesiology Department, Faculty of Dental Medicine, “Grigore T. Popa” University of Medicine and Pharmacy, 16 Universitatii Str., 700115 Iasi, Romania; ovidiu.stefanescu@umfiasi.ro; 4 Oro-Dental Diagnostics Department, Faculty of Dental Medicine, “Grigore T. Popa” University of Medicine and Pharmacy, 16 Universitatii Str., 700115 Iasi, Romania; mihaela.scutariu@umfiasi.ro

**Keywords:** periodontal disease, rheumatoid arthritis, cytokines, treatment, DMARDs, gene therapy, host modulation, inflammation

## Abstract

Rheumatoid arthritis (RA) and periodontal disease (PD) are chronic complex inflammatory diseases with several common susceptibility factors, especially genetic and environmental risk factors. Although both disorders involve a perturbation of the immune–inflammatory response at multiple levels, one major difference between the two is the different locations in which they develop. RA is triggered by an exaggerated autoimmune response that targets joints, while periodontal disease occurs as a consequence of the subgingival periodontopathogenic microbiota. Current treatment models in both pathologies involve the stratification of patients to allow therapeutic individualization according to disease stage, complexity, progression, lifestyle, risk factors, and additional systemic diseases. Therapeutic guidelines for RA comprise of five main classes of drugs: non-steroidal anti-inflammatory drugs (NSAIDs), analgesics, glucocorticoids, and disease-modifying anti-rheumatic drugs (DMARDs): biologic and non-biologic. Although various treatment options are available, a definitive treatment remains elusive, therefore research is ongoing in this area. Several alternatives are currently being tested, such as matrix metalloproteinases (MMP) inhibitors, toll-like receptors (TLR) blockers, pro-resolution mediators, anti-hypoxia inducing factors, stem cell therapy, NLRP3 inhibitors and even natural derived compounds. Although the link between PD and rheumatoid arthritis has been investigated by multiple microbiology and immunology studies, the precise influence and causality is still debated in the literature. Furthermore, the immunomodulatory effect of anti-rheumatic drugs on the periodontium is still largely unknown. In this narrative review, we explore the mechanisms of interaction and the potential influence that anti-rheumatoid medication, including novel treatment options, has on periodontal tissues and whether periodontal health status and treatment can improve the prognosis of an RA patient.

## 1. Introduction

Periodontal disease (PD) is a highly prevalent disease affecting approximately 50% of adults, with 1.1 billion people suffering from a severe form. If improperly managed, it leads to considerable loss of the tooth’s supporting tissues, and even edentulism. In this context, severe periodontal disease affects the quality of life and therefore represents an important social, healthcare, and economic concern [1].

According to Monsarrat, a total of 57 different systemic diseases have been linked to periodontal disease, and as much as one-third of recent periodontal studies explore this issue [2]. The path through which systemic diseases are linked to periodontitis is encompassed in the focal infection theory that states that oral microorganisms enter the bloodstream and travel to other organs where they can cause damage [3]. Periodontal medicine concentrates on associating periodontal pathology to other systemic illness in the light of the new biomolecular advancements in diagnosis and treatment [4].

A bi-directional relationship between rheumatoid arthritis (RA) and periodontitis has been proposed by many researchers; however, a definitive link is yet to be established. Although the presence of periodontitis has been associated to a worse rheumatoid arthritis outcome and clinical activity, not all RA patients have a more severe periodontal evolution [5]. A recent meta-analysis that included nine studies sought to assess whether non-surgical periodontal therapy could improve rheumatoid arthritis activity and concluded that RA activity was improved as assessed by disease activity score (DAS28), tender joint counts (TJC), swollen joint counts (SJC), visual analogical scale (VAS), and C-reactive protein (CRP) by non-surgical periodontal therapy [6].

Even though the early biological pathways through which the immune tolerance is affected and leads to the development and advancement of rheumatoid arthritis are unascertained, the importance of the inflammatory cascade is undisputed in the pathogenic evolution from autoimmune reaction until articular deterioration. The course of treatment in RA precisely targets specific elements of this inflammatory cascade [7]. For many years, treatment in rheumatoid arthritis has been symptomatic (steroidal and nonsteroidal anti-inflammatory analgesics), and largely non-effective, leading to joint destruction and deformation [8]. All this changed since the introduction of disease-modifying anti-rheumatic drugs (DMARDs) that have an immunosuppressive and immunomodulatory role, thus targeting the pathways through which the disease operates and evolves [9,10].

Nowadays, the treatment options for rheumatoid arthritis are diverse and the rate of novel drug development is significant; however, a definitive treatment is yet to be available. With the development and discovery of new medications, a number of other anti-inflammatory compounds have been identified, and a significant number of these could be relevant in the therapeutic management of rheumatoid arthritis and periodontal disease. This review aims to draw attention upon the treatment options in rheumatoid arthritis and assess the possible effect on periodontal tissues in order to better understand and manage both pathologies.

## 2. Ethiopathogenetic Common Ground between Rheumatoid Arthritis and Periodontal Disease

**Figure 1:**
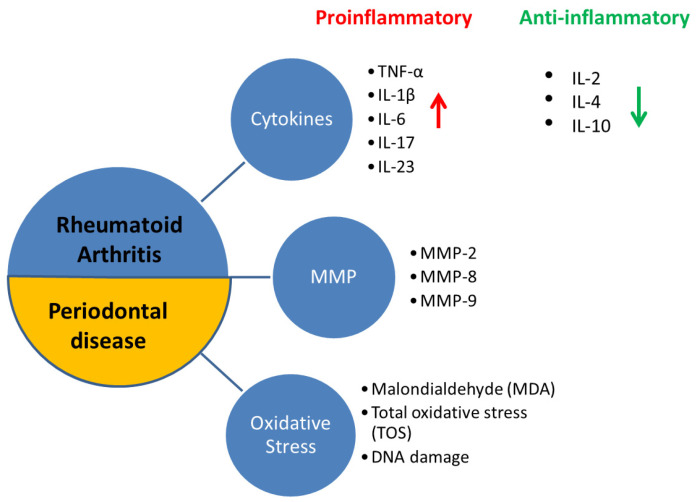
Schematic representation of the common principal molecules incriminated in the pathogenesis of periodontal disease and rheumatoid arthritis.

Rheumatoid arthritis and periodontal disease are multifaceted chronic inflammatory pathologies with numerous common susceptibility factors (especially genetic and environment factors). One important distinction between these diseases is that periodontal disease results from an exacerbated inflammatory reaction to the subgingival microbes, while inflammation in rheumatoid arthritis results from a specific exaggerated adaptive autoimmune response. Inflammation is sustained by the ongoing microbial challenge in periodontal disease and by the advancing autoimmune response in rheumatoid arthritis, building up to the increasing destruction of tissue leading to the signs and symptoms of the diseases [11]. That being said, the cytokines and biomolecules released both pathologies overlap to a staggering degree. Both pathologies display elevated values of pro-inflammatory cytokines such as tumor necrosis factor (TNF)-α, interleukin (IL)-1β, IL-6, IL-17, IL-23 but also of matrix metalloproteinases (MMP), receptor activator of nuclear factor kappa-Β ligand (RANKL), reactive oxygen species (ROS), markers of DNA damage and prostaglandin E2 [12,13,14,15,16,17,18,19,20], and low levels of anti-inflammatory cytokines such as IL-2, IL-4, IL-10, tissue inhibitor of MMPs (TIMP) and tumor growth factor-beta (TGF-β) [21,22]. This culminates in the destruction of tissues and a proinflammatory systemic state that is perpetuated in both diseases [23]. Figure 1 illustrates the common principal molecules that have been observed in the two pathologies.

Regarding periodontal risk for rheumatoid arthritis (RA) patients, an elevated risk has been reported in the literature by a recent meta-analysis that included thirteen studies [24]. Moreover, treatment for periodontal disease in RA patients has been demonstrated to diminish rheumatic disease activity [25]. Mercado et al. reported that the prevalence of rheumatoid arthritis in a group of periodontal disease subjects was six times greater than that found in individuals without periodontal disease [26]. Furthermore, after disease type stratification, there was an increased risk of incident RA and periodontal disease, and regarding duration of RA of more than 5 years, the likelihood of periodontal disease increased significantly [27]. On the other hand, a paper found no proof of an elevated risk of RA among those with severe periodontal disease [28]. Considering the common ground between the aspects of the pathogenesis of periodontal disease and rheumatoid arthritis, it is apt that researchers in the field of periodontology be familiar with the treatment options available for rheumatoid arthritis.

## 3. Current Treatment Options in Rheumatoid Arthritis and Periodontal Disease

Current treatment models in rheumatoid arthritis and periodontal disease involve stratifying patients in order to allow physicians to individualize treatment according to the complexity of the pathology, but also in terms of the systemic integration of risk assessment for each patient. Some of them display a more severe and rapidly progressive form and therefore require intensive monitoring of inflammation both locally (joints or oral cavity) and systemically, compared to patients with a less aggressive pathogenic entity [29]. The structure for the management of patients classified according to risk requires a different medical network, which integrates a myriad of specialties related to the specific pathology in question. It also requires an integrated network of practitioners that allows the generation of current data on epidemiology, the accuracy of diagnosis methods and the validity of treatments, whose ultimate goal is to develop protocols and evaluate the success of treatment. Moreover, for the majority of rheumatoid arthritis cases a combination of several drugs is employed, as until now there is not one treatment that manages all the symptoms of these patients and can provide a definitive cure [30].

For the treatment of rheumatoid arthritis, several classes of anti-inflammatory drugs have been proposed, some of them also considered as candidates for periodontal disease therapy by modulating host response, including: corticosteroids, DMARDS (disease-modifying antirheumatic drugs), NSAIDs (non-steroidal anti-inflammatory drugs), MMP (matrix metalloproteinases) inhibitors, clinically modified tetracyclines, anticytocins and biologic therapies, lipid mediators of inflammation resolution, small-molecule compounds, histone deacetylase inhibitors, RANKL (receptor activator of nuclear factor kappa-Β ligand) inhibitors, bisphosphonates, toll-like receptor inhibitors, as well as the combined antibacterial and anti-inflammatory approach [31].

### 3.1. Corticosteroids

Corticosteroids represent a class of therapeutic agents similar to cortisol, which have a broad applicability in medicine, mainly due to their anti-inflammatory and immunosuppressive characteristics. They hinder the generation of prostaglandins and leukotrienes, suppress the cell-mediated immune response which leads to a diminished T cell proliferation by suppressing gene transcription of various interleukins and gamma interferon, and downgrade the humoral response by simultaneously reducing B cell counts and antibody production [32]. Furthermore, they stabilize the lysosome membrane, increase the permeability of blood vessels, inhibit vasodilatation and proliferation of fibroblasts, and promote osteoclastogenesis [33].

A study that analyzed the effect of inhaled corticosteroids on a group of patients with chronic obstructive pulmonary disease concluded that this therapy may affect bone metabolism and cause a reduction in bone mineral density of the maxillary bones [34]. These observations were enforced by a meta-analysis that states that patients with asthma treated with corticosteroids have a higher prevalence of periodontitis compared to subjects under another therapeutic regime [35]. On the other hand, short-term use of corticosteroids might be beneficial for periodontal tissues; a study highlighted that prednisone, with or without the association of surgical therapy, was efficient in reducing inflammation periodontal parameters and salivary cortisol level in patients with chronic periodontitis [36].

Due to the fact that steroids are associated with a multitude of side effects, the risk-benefit outline is disadvantageous in the context of periodontal treatment and therefore caution must be employed when patients are under this treatment

### 3.2. NSAIDs (Non-Steroidal Anti-Inflammatory Drugs)

NSAIDs are used in a broad number of inflammatory and painful diseases including autoimmune diseases and various painful conditions. Common side effects include gastrointestinal symptoms, hemostatic effects, hypersensitivity reactions, renal effects, as well as headache and vertigo. Prostaglandins are efficacious proinflammatory mediators relevant to the progression of periodontal disease and are produced by a multitude of periodontal cell types (epithelial cells, macrophages, neutrophils, and fibroblasts) as a response to lipopolysaccharide release [37]. 

Since NSAIDs inhibit prostaglandin synthesis, these drugs have been proposed in order to reduce the progression of periodontitis. Numerous studies have shown that patients who use NSAIDs for long periods of time have reduced probing depth, decreased alveolar bone loss rate, and less gingival inflammation than control groups [38,39,40].

### 3.3. MMP Inhibitors (Matrix Metalloproteinases)

MMPs are enzymes that degrade a wide number of matrix extracellular proteins, along with collagen. They are at the forefront of tissue degradation, including articular and periodontal, and are produced by various cell types: macrophages, neutrophils, fibroblasts, keratinocytes, osteoclasts, and endothelial cells [41]. RA is defined by progressive joint destruction with cartilage and bone loss and aggressive activation of synovial fibroblasts that have a tumor-like aspect. Rheumatoid arthritis synovial fibroblasts secrete matrix-degrading enzymes, among which are MMPs, that promote destruction of cartilage in damaged joints of RA subjects [42]. Tumor necrosis factor-α (TNF-α), interleukin (IL)-1β, IL-6 stimulate MMP genes by ligating various factors such as activator protein-1, mitogen-activated protein kinase, and nuclear factor-κB (NF-κB) to their promoters [43,44,45,46]. Dysregulation of epigenetic modifications, like DNA methylation, histone modifications, and microRNA (miRNA) signaling determine the activation of MMP genes in synovial fibroblasts. The cartilage can be directly harmed by some proteinases such as matrix metalloproteinases, cathepsins, neutrophil elastase, and ADAMTS (a disintegrin and metalloproteinase with thrombospondin motifs) [44]. Matrix metalloproteinases are believed to have a crucial position in the tissular degradation in arthritic joints, because expression of MMP-1, MMP-3, MMP-9, and MMP-13 is upregulated in rheumatoid arthritis synovial fibroblasts [47]. Moreover, serum MMP-1 and MMP-3 parallel disease activity and predict radiological progression, whereas disease activity is significantly reduced during conventional and biologic DMARDs therapy, and this is mirrored by a downregulation of the same metalloproteinases [48,49]. In a collagen-induced arthritis model, administration of a MT1-MMP selective inhibitory antibody inhibits both cartilage deterioration and disease progression, demonstrating noteworthy therapeutic capabilities [50,51]. 

Although a variety of MMP inhibitors have been analyzed, many did not have the expected results in clinical trials or are still in the development stage [52]. Newer small MMP inhibitors are now being tested in the hope of an improved efficacy [53]. For example, Trocarde™, a collagenases (MMP-1, MMP-8, and MMP-13) selective inhibitor, is presented as having no serious adverse events and is well tolerated by recipients [54]. It showed promise as a therapeutic agent for RA subjects given the results of animal models; nonetheless, the drug failed to hamper the advancement of articular damage arthritis subjects in phase III trials [53]. MMP inhibitor specificity has been low because they target the catalytic sites of enzymes; however, not all MMPs are harmful; for example, MMP-2 and MMP-8 displayed protective effects in RA [55,56]. Besides binding site specificity of MMPs, intelligent drug delivery systems (that signalize the specific position of metalloproteinase inhibition directly) are another noteworthy approach to diminish off-target secondary effects. Presently, three drug release methods that are MMP-responsive have been advanced: structure disassembly, linker cleavage, and membrane “uncorking”. Another process may be the encapsulation of highly selective mAb inhibitors within MMP-responsive nanoparticles that can be administrated orally [57]. Future researches should concentrate on working with specific signal peptides and structure binding sites in order for MMP therapies to have clinical success.

The main MMPs associated with collagen destruction in the periodontium are MMP-8 and 9 secreted by large numbers of neutrophils in affected periodontal tissues. The anti-MMP capabilities of tetracyclines were initially observed in Minocycline, but Doxycycline is the most effective for this purpose [58]. Properties that are relevant in the treatment of periodontal disease are the inhibition of active but also latent MMP, the inhibition of reactive oxygen species and the reduction of osteoclastic activity and bone resorption [59]. By using the sub-antimicrobial dose (20 mg/2/day for 3 months) as a host response modulator, the goal is to reduce the pathological level of MMP associated with periodontal disease but not to completely inhibit it, as MMP play a vital role in multiple pathological processes, but also in physiological ones [59]. Doxycycline is prescribed as an adjunct to conventional non-surgical treatment in order to increase the resolution of inflammation. Its use is contraindicated in individuals with a history of allergy to tetracyclines, during pregnancy or in children [59]. A systematic review observed a statistically significant difference in attachment gain in favor of Doxycycline use compared to placebo. The reduction in probing depth for sites larger than 7 mm was 1.77 mm in the placebo group (non-surgical therapy only) and 2.31 mm in the Doxycycline group. Researchers have raised the issue of statistical relevance related to clinical improvements, and even though it is not possible to forecast the exact advantage of this medication in a specific patient, studies suggest clinically relevant improvements that can be achieved after Doxycycline therapy [60]. In a study by Prashaw, 43% of sites with an initial probing depth greater than 6 mm revealed a 2 mm reduction in the placebo treated individuals, in comparison to 62% of sites in Doxycycline subjects [61]. The issue whether these patients can develop antibiotic resistance has also been raised, but there is no evidence with regard to the administration of subantimicrobial doses of Doxycycline on the subgingival or intestinal microbiota, even when administered for long periods of up to 9 months [62]. An intriguing alternative to doxycycline could be chemically modified tetracycline analogues, because they do not have any antibiotic attributes but still maintain anti-MMP characteristics. One of the most studied was the chemically modified tetracycline 3 (6-demethyl-6-deoxy-4-dimethylaminotetracycline), a suppressant of MMP activity and cytokine production [63]. These compounds were shown to diminish the collagenolytic activity generated by *Porphyromonas gingivalis,* reduce the secretion of proinflammatory cytokines, and inhibit alveolar bone loss [64].

The use of Doxycycline was proven as safe and its application results in an augmented clinical outcome when utilized as adjunctive therapy, but it is not clear exactly in which cases it should be used, namely, in all cases of periodontal disease (as suggested by pharmaceutical companies) or in a more individualized manner, focused on the patient’s biological needs and particularities. One must take into account the compliance of patients who, in addition to the demands related to the treatment of periodontal disease, must follow a daily drug treatment for at least three months, emphasizing once again the need for a treatment centered on the patient’s needs.

Certainly, further research is needed to explore the efficacy of chemically modified tetracyclines in modulating inflammation and diminishing connective tissue damage in periodontal disease and rheumatoid arthritis, but also in metastases and metabolic disorders of the bones [65]. Even though there has been a new found interest in MMP inhibitors brought on by recent discoveries in biochemical functions and molecular pathways, doxycycline still is the only drug in this class recognized by the US Food and Drug Administration for the treatment of diseases, wherein an elevated level of MMPs determine the onset and progression of certain diseases. As such, the therapies discussed could be an important future addition to the treatment of both diseases.

### 3.4. DMARDs

The term DMARDs is utilized in order to refer to a class of therapeutic agents that are not generally related, but this term is used in order to differentiate them from:

a. NSAIDs that diminish the inflammatory process but do not address the cause of RA;

b. Steroids that diminish the immuno-inflammatory response but have no effect on the advancement of the disease.

That being said, NSAIDs and steroids are useful in controlling symptoms in RA, but only DMARDs influence disease progression [66]. As a class of drugs, they are prescribed in the treatment of inflammatory diseases, mainly rheumatoid and psoriatic arthritis and ankylosing spondylitis, but also inflammatory myositis, vasculitis, Crohn’s disease, cancer, Sjogren syndrome, systemic lupus erythematosus, and systemic sclerosis, to name a few [67]. They have an immunomodulatory and immunosuppressive effect and are classified as conventional DMARDs or biologic DMARDs. The former do not act specifically on certain pathways of the immune system; rather, they have a dampening effect on it, acting non-specifically and over a long period of time by diminishing pathological processes within RA [68]. During treatment, a reduction in C-reactive protein, rheumatic factor (RF), and sedimentation rate of erythrocytes should be observed, and also a downscaling of the damage to bone and cartilage [8].

Biologic DMARDs were introduced more recently in the treatment of rheumatoid arthritis and are, for the most part, prescribed after conventional DMARD therapy is unsuccessful (continuing disease activity or progression of disease determined clinically or radiographically). They are sometimes called “targeted biologic agents” that are produced by means of molecular biology (recombinant DNA) techniques [68]. Biologics target a specific pathway of the immune system and are highly specific, being designed to prevent or reduce the inflammation that damages joints. These agents specifically address molecules of immune system cells, joints, and the byproducts that are secreted that can determine and perpetuate inflammation and joint destruction. There are many types of drugs in this category, according to a particular type of molecule involved in this process. A few of these therapeutic agents are monoclonal, chimeric humanized fusion antibodies, whilst others are receptors that were merged to a part of the human immunoglobulin or small molecules such as Janus kinase (JAK) inhibitors [69].

DMARDs are potent systemic modifiers that can have various unwanted side effects such as nausea, skin rashes, oral ulcers, digestive symptoms, and others. Moreover, they act as suppressants of the immune system, which can lead to major unwanted potentially life-threatening conditions, like tuberculosis reactivation, or an increased risk of malignant tumors development [8].

In RA therapeutic strategies, biologics are frequently associated with methotrexate in individuals with little response to conventional DMARDs. Biologics encompass various anti-cytokine drugs that impede the activity of specific cytokines. TNF-α is a principal target in the advancement of such therapies in the management of arthritis. Other cytokinic targets involve IL-1, IL-6, IL-17, IL-15, and, more recently, IL-23 [68].

When considering biologics as potential therapeutic agents in the treatment of periodontal disease, animal studies have shown a potential effectiveness in diminishing alveolar bone loss and reducing inflammatory cell infiltrate [70]. The biological rationale for utilizing these agents as modulators of periodontal disease expression in vitro has been proven by studies on mice with TNF-α receptor deficiency that had diminished bone loss and inflammatory response (reduced periodontal inflammation) after *A. actinomycetemcomitans* inoculation [71]. 

Currently, the majority of research that assess the utilization of anti-cytokine agents for periodontal disease in human subjects comprise of small clinical trials that evaluate the periodontal status in RA patients. The conclusions drawn from these studies are inconclusive, most likely due to the limited number of patients, with various periodontal pathological entities, chosen for the anti-cytokine RA therapy, indiscriminately of their periodontal condition. Case in point, a study that assessed the effect of intravenous anti-TNF-α (infliximab) treatment in RA subjects identified increased gingival inflammation; however, the probing depth remained unmodified over time [72]. On the other hand, another study found that RA subjects under infliximab therapy had diminished scores on bleeding on probing, lower measurements of probing depth, and reduced concentrations of TNF-α in the crevicular fluid, when compared to patients with rheumatoid arthritis who did not receive infliximab or non-arthritis controls [73].

Another study of three groups of subjects (one with autoimmune diseases including rheumatoid arthritis without anti-TNF-α treatment, the second with rheumatoid arthritis who received anti-TNF-α treatment, and a healthy control group with no autoimmune diseases and no anti-TNF-α treatment) found that the patients with autoimmune disease who did not receive anti-TNF-α treatment had more gingival inflammation, bleeding on probing, higher mean probing depth, and higher concentrations of TNF-α in the crevicular fluid than in patients in the other two groups [74]. The results of this study highlight that patients with autoimmune diseases have increased periodontal inflammation when compared to subjects without autoimmune disease, and that anti-TNF-α treatment reduces inflammation in periodontal tissues.

*P. gingivalis* autoantibodies have been substantially correlated with the presence of RA autoantibodies (rheumatoid factor and anti-citrullinate protein antibodies-ACPA) in individuals classified as high risk of RA development. This suggests an important role of *P. gingivalis* in the early loss of autoantigen tolerance that occurs in this rheumatic pathology [75]. So far, no prospective study has evaluated anti-cytokine drugs as a specific therapy for periodontal disease. Nonetheless, periodontal status was assessed in subjects at baseline and after the initiation of anti-cytokine therapy in RA [76]. The periodontal indexes (plaque amount, probing depth, gingival bleeding, and loss of attachment) were not significantly modified 6 months after treatment, although an improvement was noted in the parameters of the severity of RA activity (C-reactive protein and erythrocyte sedimentation rate values, disease activity score) during this time. Moreover, improvements in RA parameters favored non-periodontitis individuals, while patients who had periodontal disease did not show significant improvements [76]. 

The type of DMARD may affect the presence of certain periodontopathogenic bacteria [77,78]. Moreover, a group of researchers observed an antimicrobial activity for certain drugs such as a broad-spectrum antimicrobial action for sulfasalazine and aurothiomalate and an activity against *Fusobacterium nucleatum* for Methotrexate [79]. This is an interesting observation and it draws attention to the importance of a healthy oral environment and how certain medication can modulate the bacterial ecology even though it was prescribed for a non-oral cavity pathology.

A study by Romero-Sanchez assessed the outcome of anti-rheumatic therapy, both conventional and biologic, on rheumatoid arthritis subjects on their periodontal status. Anti-TNF-α was correlated with a higher *T. denticola* count; however, the combined therapy with methotrexate yielded a lower severity of clinical attachment loss; methotrexate plus leflunomide, however, determined higher values of this particular index. The type of antirheumatic therapy influenced the presence of *T. forsythia, E. nodatum*, and *P. gingivalis*; moreover, higher anti-citrullinated protein antibodies (ACPAs) values were associated with the presence of periodontopathogens from the red complex [77]. 

In a study that assessed the effect of anti-rheumatic therapy on the periodontal status of patients, observed that subjects treated with a combination of metotrexate and anti-TNF-α had higher bleeding on probing values when compared to leflunomide alone and metotrexate combined with rituximab [78]. Furthermore, several periodontopathogenic bacteria (*Porphyromonas gingivalis, Fusobacterium nodatum, Treponema denticola*, and *Capnocytophaga species*) were associated with certain treatment subgroups [78]. On the other hand, another study has contradictory results and states that metotrexate and anti-TNF treatment has an insignificant effect on the periodontal status of RA subjects. [80].

Janus kinase inhibitors (JAKi) are a newer promising addition to DMARDs and are classified as targeted synthetic small-molecule compounds. They have proved clinical efficacy when compared to classically administered DMARDs; however, long-term data is still not available in the literature regarding their behavior [81]. In vitro, tofacitinib, upadacitinib, and baricitinib were shown to inhibit lymphocyte activation and proliferation in peripheral blood mononuclear cells. Moreover, tofacitinib and baricitinib indicated capabilities of DNA damage repair [82].

The expression of janus kinase (JAK)/signal transducer and activator of transcription (STAT) is modulated by *P. gingivalis,* objectified by the production of prostaglandin E2 and cyclooxgenase-2, and elevated the protein expression of JAK/STAT induced by lipopolysaccharide and nicotine. The addition of a JAK inhibitor blocked cyclooxgenase-2 and prostaglandin E2 production, but also the expression of TNF-α, IL-6, and IL-1β in osteoblasts stimulated with lipopolysaccharide and nicotine [83]. Tofacitinib was shown to ameliorate periodontal inflammation along with a decrease of IL-6, TNF-α, anti-cyclic citrullinated peptide immunoglobulin G and MMP-3 [84]. That being said, more research is necessary to comprehend the relationship between periodontitis and JAKi therapy.

Thus, certain biologics may have a protective effect against periodontitis and may slow down its progression by affecting key cytokines of the proinflammatory cascade and limiting bone loss. On the other hand, other compounds such as Vitamin D may have a potential protective role in both pathologies, as a low concentration was observed in rheumatoid arthritis patients with severe periodontitis (stage IV) compared to moderate PD (stage II) [85].

### 3.5. Lipid Mediators of Inflammation Resolution

A fundamental constituent of the inflammatory response is the resolution of inflammation. It was previously thought to be characterized by a passive progressive decrease in inflammatory processes, as soon as the initial catalyst was removed. However, nowadays it is accepted that acute inflammation resolution is an exceedingly regulated process, and certain endogenous “pro-resolution” mediators promote leukocyte infiltration remission and elimination by apoptosis, increase vascular permeability and vasodilation and removal of necrotic tissue and pathogens [86]. Inflammation resolution is vital for the prevention of chronic inflammation; however, if this process does not occur, then chronic inflammation commences, causing tissue damage due to the perpetuation of injuries. Molecules that act as signals to stop inflammation are appealing targets in the management of inflammatory diseases, like periodontitis and rheumatoid arthritis, and could potentially have an edge over classic anti-inflammatory drugs that have significant undesirable side effects [87]. 

A category of therapeutic compounds that were advanced for this purpose are resolvins, protectins, and lipoxins. Resolvins and protectins derive from omega-3 fatty acids, while lipoxins derive from arachidonic acid [88,89]. Through identifying these signal molecules of the final stages of the inflammatory cascade and their application as therapeutic agents, a broader understanding of the evolution of chronic inflammatory diseases would be possible. These inflammation-resolving and immune-regulatory pathways are an appealing alternate option to tackle inflammation in rheumatoid arthritis. Strategies to regulate dysfunctional immune pathways are particularly compelling in established disease in order to prevent relapses, which can occur due to dose reduction of therapeutics, and this is particularly important for the induction of long-term remission. Regarding the anti-inflammatory cytokines that have a regulatory function which are displayed by RA subjects, they point towards a type 2 immune response. Up-regulating particular cytokines, such as IL-4, IL-5, IL-9, IL-13, and IL-33, could cause allergy-like responses in some subjects; however, in most cases, an increased genetic susceptibility is most likely the cause [21]. Delivery of these cytokines through a therapeutic medium or augmentation of their expression may be a viable option.

Another option for the activation of type 2 immune-regulatory responses is the delivery of peptides such as helminth-derived compounds and synthetic ES-62 small-molecule analogues, which have been observed experimentally to inhibit arthritis [90]. These products have a protective effect through an initiation of a type 2 response that down-regulates TH17 lymphocytes and up-regulates B cells that secrete IL-10 [91]. A peptide that possesses this type of immune-regulatory properties is FhHDM-1, which favors the differentiation of regulatory phenotype macrophages and inhibits the generation of IL-6 and TNF [92].

After the source of immune activation is eliminated, a pro-resolution process is initiated and pro-resolving mediators are produced. Their main biological activity is to inhibit the synthesis of adhesion molecules in endothelial and epithelial cells. They lower the generation of IL-6, IL-8, and monocyte chemoattractant protein-1, dampen the production of reactive oxygen species, and drive towards an anti-inflammatory M2 phenotype [93]. The level of pro-resolving lipid mediators Lipoxin A4, Resolvin E1, and Resolvin D1 was significantly lower in active RA when compared to healthy or in remission individuals [94]. 

RA is outlined by a disregulation of the lipid metabolism that manifests as a modified profile of fatty acids in tissues and synovial fluid, and since dietary fatty acid supplements can alleviate the symptoms of RA patients, eicosanoids and polyunsaturated fatty acid-derived pro-resolving lipid mediators have been a highlight of recent research [95]. Extracellular vesicles have been regarded as most attractive as they have the capability of repairing damaged cartilage in animal models [96].

In vitro studies suggest that these molecules might play a substantial part in resolving periodontal inflammation. In a model of experimental periodontal disease induced by *P. gingivalis* in animals, the local delivery of resolvin E1 led to the resolution of inflammation and anatomical reestablishment of periodontal tissues (soft and hard) when compared to the control group, objectified histologically through the absence of osteoclasts, as well as regeneration of periodontal tissues [97]. Balta et al. observed that resolvin E1 caused a reduction in alveolar bone loss and an elevated osteoprotegerin expression, regardless of RANKL, indicating that this molecule has a direct osseous remodeling modulatory effect and leads to the restoration of an optimum osteoprotegerin–RANKL ratio [98]. There have been a limited number of studies that explore pro-resolution mediators in human subjects, and this is an emerging research area. A study analyzing dietary supplementation with low dose acetylsalicylic acid and fish oil as adjuncts to conventional non-surgical periodontal treatment observed greater reductions in probing depth and RANKL and MMP-8 synthesis levels in patients receiving combination adjuvant therapy compared to control groups receiving only non-surgical therapy [99].

### 3.6. Small-Molecule Compounds

Recently, there has been increasing interest in a number of small-molecule compounds targeting pathways that are mediated by specific cytokines. In this category, histone deacetylase inhibitors are among the most studied molecules. An important advantage of these compounds is their suitability to local administration—therefore, compliance would be improved. The epigenetic control of gene expression is accomplished by the covalent conversion of chromatin, and this includes histone acetylation and methylation of DNA. These operations alter transcription factors access to DNA coding sections, thus influencing the expression of genes and generation of proteins [100]. Two enzymes control histone acetylation: histone deacetylases and histone acetyltransferase [101]. Research on histone deacetylase inhibitors performed in various chronic inflammatory and malignant diseases has highlighted their potential use as anti-inflammatory agents [101]. This has led to the recognition of the potential anti-inflammatory attributes of these molecules, possibly by inhibiting the nuclear-factor KappaB pathway and subsequently suppressing the inflammatory cytokines, as well as inhibiting the expression of matrix metalloproteinases [102]. Considering the fact that studies have described both pro-inflammatory and anti-inflammatory effects of these molecules, clearer research is needed to fully clarify their indications and biological properties [103].

Histone deacetylase inhibitors illustrate impressive therapeutic potency in arthritis animal models and dampen proinflammatory cytokine production in synovial tissue. They suppressed IL-6 generation by fibroblast-like synoviocytes induced by TNF-α, IL-1β, and Toll-like receptor ligands, but also promoted the decay of IL-6 RNA messenger in synoviocytes and macrophages. Furthermore, a role in the regulation of fibroblast-like synoviocytes production of IL-8 and MMP-1 was observed, thus successfully inhibiting inflammation in RA [104]. A particular compound called givinostat (ITF2357) diminished the expression of IL-1β-inducible transcripts (IL-6, IL-8, MMP-1 and ADAMTS1, chemokines CXCL2, CXCL5, CXCL6, CXCL10, matrix-degrading enzymes, and other inflammatory mediators). Moreover, it accelerates the mRNA degradation of Prostaglandin-Endoperoxide Synthase 2, CXCL2, IL-6, and IL-8 [105]. A selective histone deacetylase 6 inhibitor, CKD-L, significantly inhibited IL-1β and TNF-α, and increased IL-10 [106], while CKD-506 had an inhibitory effect reducing IL-6, IL-8, MMP-1, and MMP-3 production by activated fibroblast-like synoviocytes [107]. In animals (rats), orally administered CKD-506 ameliorated clinical arthritis activity in a dose-dependent manner, and when administered in combination with methotrexate, they presented a synergistic effect [107]. All these studies highlight the anti-inflammatory properties therapeutic potential of histone deacetylase inhibitors in the treatment of RA.

An interesting property of some histone deacetylase inhibitors with implications in periodontal disease pathogenity is the fact that they can modulate RANKL effects on inflammatory cells and osteoclasts, leading to the inhibition of osteoclastic activity induced by RANKL in macrophages, probably due to the reduced expression of osteoclastic transcription factors, such as the TNF receptor, activated T cell nuclear factor, and osteoclast-associated receptor. In a murine model of experimental periodontal disease, topical application of 1179.4 b (a novel histone deacetylase inhibitor) produced an important decline of alveolar bone loss induced by *P. gingivalis*, even though the histone deacetylase inhibitor did not diminish inflammation [101]. The ramifications of these findings are that these compounds can be used as new therapeutic agents in the management of pathologies characterized by bone loss, such as periodontal disease. Previous studies demonstrated a safe profile and good tolerability, as well as clinical effectiveness. Nevertheless, potential side effects should not be ruled out, especially when considering the large-scale effects of these compounds in cells and tissues [108]. On the other hand, as a means to reduce the potential side-effects, topical administration could be employed, or advancement of specific inhibitors that target certain particular pathological pathways.

### 3.7. RANKL Inhibitors

Receptor activator of nuclear factor κ B (RANK) is a member of the tumor necrosis factor receptor (TNFR) molecular sub-family and is the cell surface receptor of RANK-Ligand (RANKL). RANK is expressed by dendritic cells, osteoclast precursors, mature osteoclasts, and many other cell types. RANKL binds to RANK and is expressed by fibroblasts, bone marrow stromal cells, osteoblasts, and T and B lymphocytes, to name a few. When RANKL binds to RANK this leads to the differentiation and activation of osteoclasts, and subsequent bone resorption. Osteoprotegerin (OPG), on the other hand, is a decoy receptor for RANKL that modulates the RANK signaling pathway by competing for RANKL, thus inhibiting osseous tissue resorption. The RANK/RANKL/osteoprotegerin signaling pathway has a key role in the regulation of bone metabolism, and various cytokines can increase the RANKL–osteoprotegerin ratio, amplifying bone loss [109]. Denosumab, an anti-RANKL monoclonal antibody, is utilized as a therapeutic agent in bone resorptive pathologies, such as osteoporosis, as it leads to a progressive increase in bone mass, although its precise mechanism of action remains controversial [110]. Although denosumab can inhibit the advancement of joint destruction when used in conjunction with conventional or biological DMARDs, this without added adverse effects, it has little or no effect regarding disease activity or destruction of cartilage and is used predominantly in the early stage of rheumatoid arthritis [111,112].

To date, there are few reported studies of denosumab treatment in the context of periodontal disease, the majority being case reports. There is only one case reported in the literature of osteonecrosis of the jaw in a patient with osteoporosis treated with denosumab that underwent non-surgical periodontal therapy, so incidence and possible preventive measures are still unknown at this point [113]. Another study showed a statistically significant correlation between denosumab-related osteonecrosis of the jaw and periodontal disease in a group of patients with stage IV solid cancer with bone metastases treated with denosumab [114].

In a periodontitis mice model that compared denosumab with zoledronate, administration of the former substantially repressed the destruction of alveolar bone and exposure of tooth root, while the latter only slightly inhibited alveolar bone destruction and did not repress root exposure. The authors indicate that denosumab may be a possible candidate for preventing the destruction of alveolar bone correlated with periodontal disease [115]. Still, caution should be employed when treating these patients, as there have been reported cases of osteonecrosis of the jaw after denosumab treatment in the context of oral surgery [116,117].

### 3.8. Bisphosphonates

Bisphosphonates are compounds that alter the activity of osteoclasts and inhibit osseous resorption; the main mechanism of action is by inducing osteoclast apoptosis. Bisphosphonates are widely utilized in the treatment of resorptive bone pathologies, but have been recently discussed as an addition in the treatment of RA due to their role in inhibiting focal joint destruction; furthermore, they act as inhibitors of IL-1, IL-6, and TNF-α [118].

In a clinical study in RA patients with secondary osteoporosis, a group of researchers that analyzed the benefit of adding zoledronic acid to methotrexate versus methotrexate alone, observed a significant improvement in the global disease activity, diminished fracture risk, and an improved quality of life [119].

A number of clinical trials have been initiated, evaluating the application of bisphosphonates in the treatment of periodontal disease. Even though the initial results seemed promising, as small-scale studies have demonstrated an improvement, this was not confirmed by more ample clinical trials. Moreover, the occurrence of undesirable side effects (osteonecrosis related to jaw medication) characterized by the exposure of the necrotic bone and increased infection-risk, especially after oral surgery, placed this line of research on a closed path for now [120]. Considering the possibility of occurrence of this serious pathology, bisphosphonates utilization as an adjunctive treatment for periodontitis is not recommended.

## 4. Future Research Directions and Potential Risks

Host response modulation therapy is an established treatment strategy, and research has put forward a considerable number of drug candidates which might have an important role in the management of periodontal disease. Anti-cytokine therapeutic agents have a demonstrated efficacy in rheumatoid arthritis management, and considering the ethiopathogenic common ground between this rheumatic pathology and periodontal disease, it is natural for researchers to be interested in anti-cytokine therapies as potential modulators of the host response in the treatment of periodontal disease. Similar to other chronic inflammatory or autoimmune diseases, our knowledge of all cytokine functions in periodontal disease is nowhere near exhaustive, and the simple inhibition of a specific cytokine will not necessarily entail a clinically significant outcome on the considered pathology. Anti-TNF-α therapy in patients with rheumatoid arthritis leads to the reduction of other pro-inflammatory mediators, such as IL-1β and IL-6, causing an improvement in the anti-inflammatory effect [121]. Even so, the use of these therapies does not lead to the comprehensive elimination of the pathology; thus, a favorable outcome is verified by compound scales that pay regard to the percent improvement of the clinical situation and symptoms of the disease. Besides the increased cost of these therapies and significant possible side effects, the mode of administration (subcutaneous of intravenous injections) is also a limiting factor in their administration in periodontitis. 

Small-molecule compounds, such as histone deacetylase inhibitors, show real potential in the management bone resorptive pathologies, and the possibility of a synergetic therapy with anti-inflammatory drugs that minimizes bone resorption along with inflammation is intriguing. Nevertheless, one should not minimize the potential domino effect of using these inhibitors on other vital molecular and cellular processes; thus, supplementary research is recommended to assess the risk–benefit profile [122]. On the other hand, pro-resolution lipid mediators have a major selling point, which is that they are generated endogenously as a natural physiological response to inflammation with the purpose of resolving it, as opposed to inhibitors which act more aggressively and could interfere with the host’s defense system [123]. The activation of inflammasomes has been associated with RA onset and progression due to their upregulation of several pro-inflammatory cytokines and chemokines [124]. Until now, there have been several inflammasomes identified, of which the nucleotide binding oligomerization domain and leucine rich repeat pyrin 3 domain (NLRP3) are the most researched. The NLRP3 inflammasome is a key constituent of the innate immune system and has a crucial role in infection recognition and promoter of autoimmune responses [125]. Moreover, it is principally expressed in neutrophils, dendritic cells, and macrophages, coupled with an increased expression of RNA messenger and increased protein generation related to the NLRP3 inflammasome in RA subjects [126,127]. In this context, several studies have observed beneficial effects of natural products that inhibit NLRP3, such as Licorice-processed Daphnes Cortex, Galangin and Cinnamaldehyde on RA activity [128,129,130].

An interesting alternative may be represented by the addition of therapeutic agents that involve targeting aspects of Toll-like receptor expression, since they may involve heterogeneous pathways of the inflammatory response. Toll-like receptors (TLR) are protein recognition receptors that offer an immune response after coming into contact with pathogen-associated microbial patterns and danger-associated molecular patterns (DAMPs) that are produced as a result of cellular injury [131]. The TLR pathway and its analysis have granted new insights into the mechanisms and pathogenesis of rheumatoid arthritis and periodontitis.

Hydroxychloroquine, a therapeutic agent administered in RA has been shown to significantly suppress human B cell and also dendritic cells functions mediated by TLR9 during the inflammatory processes [132]. The mechanism of action through which hydroxychloroquine acts in rheumatoid arthritis is not yet fully known; thus, it is interesting to explore the potential implications of a combined therapy with TLR modifiers.

Blocking the activation of TLRs during RA progression was reported to have a dampening effect on pathogenesis. TLR4 inhibitor TAK-242 inhibited the elevated expression of MMP-1, IL-6, IL-8, and VEGF, and it also markedly improved inflammatory symptoms of joints 21 days after treatment, according to histology and RT-PCR [133]. It appears that this molecule blocks a specific signaling pathway and displays potential as a novel treatment in rheumatoid arthritis [133]. Another potential drug candidate is NI-0101 (a humanized monoclonal antibody) that blocks TLR4, confirmed to inhibit cytokine release induced by lipopolysaccharides. In a clinical trial of ACPA-positive subjects with inadequate response to initial methotrexate therapy, by using NI-0101 alone, no response was observed; thus, monotherapy does not ameliorate disease parameters and successful management may require more ample or earlier inhibitory treatment [134].

We can observe that studies are currently ongoing to advance molecules that activate Toll-like receptors, but also antibodies that would exert an inhibitory effect, with the aim of developing new agents that could be used in the management of a number of pathologies, including periodontitis, cancer, rheumatoid arthritis, inflammatory diseases of the gastrointestinal tract, and other autoimmune diseases. However, the research is in its infancy, and ethical and patient safety concerns are substantial, considering the fundamental importance of these receptors in the inflammatory process and in immunity. 

### Lessons Learned from Rheumatoid Arthritis Treatment-Combined Approaches for Periodontitis Treatment

As previously mentioned, inflammation of the periodontal tissues is initiated and sustained by periodonto-pathogenic bacteria, and much of the tissue degradation is a result of the exaggerated inflammatory riposte of the host. Thus, similar in principle to how rheumatoid arthritis therapy is carried out, a combination of therapeutic agents that target both inflammation and the microbial assault could prove to be a highly successful therapeutic strategy. An excellent example of this principle is the use of azithromycin, a synthetic antibiotic derivative of erythromycin, that has an antimicrobial bacteriostatic action. At the same time, similar to other macrolides, it also has anti-inflammatory properties that inhibit the generation of proinflammatory cytokines, namely, IL-1β, IL-6, TNF-α, and granulocyte-macrophage colony stimulating factor, as well as various chemokines [135]. Its use as an antimicrobial adjuvant in conventional non-surgical periodontal therapy has been evaluated in some studies, with evidence of clinical benefit, and further research in this direction should further clarify the real clinical benefit [136].

Due to the fact that the exacerbated inflammatory response is regarded as an essential etiopathogenic element, exaggerated inflammation and inadequacy to resolve inflammation may affect the outcome of the disease. In the past, research was concentrated on discerning the contribution of leukotrienes and prostanoids in advancing the inflammatory cascade for the purpose of manipulating it; however, nowadays the focus has shifted to increasing inflammation resolution by increasing the “off” signal and encouraging healing and microbial clearance [137].

Bone regeneration achieved through the utilization of biological mediators encompasses growth factors, cells, and gene therapies. Administration of stem cells is still a young, insufficiently explored therapeutic domain and many obstacles remain in their implementation, in terms of safety and regulation. The usual growth factors that are investigated are bone morphogenetic proteins, platelet-derived growth factors, and molecules involved in vascular and cellular growth [138]. Gene therapy that uses plasmids in order to introduce the suitable genes within specific cells is being evaluated and is considered safer than viral vectors, which would have unpredictable long-term effects [139]. Some preclinical studies that use mesenchymal stem/stromal cell therapy indicate that patients could benefit the most out of this treatment modality if it is applied early, in the first phases of inflammation [140].

Development and discovery of drugs is costly and time-consuming. Rebranding and repurposing of drugs, on the other hand, is extremely advantageous and desirable. Various drug classes and molecules are being investigated for their immunomodulatory properties in order to evaluate their applicability in the treatment of periodontal disease with relation to rheumatoid arthritis [141]. The challenge remains to capitalize on information from fundamental and animal studies in order to further advance and assess these compounds as novel therapeutic agents, and to evaluate the clinical risk–benefit profile as well as the possible variations in response and interactions with other drugs and potential adverse effects at the individual and population level.

## 5. Conclusions

The effect of anti-rheumatic drugs on the periodontium is still largely unknown as the majority of these therapies are still being tested and are new to the treatment protocols. Nevertheless, in the near future, it is likely that new and effective anti-inflammatory and modulating pro-resolving therapies will emerge in the treatment of both chronic inflammatory diseases.

## Data Availability

Data sharing not applicable.
